# Olanzapine-Induced Rhabdomyolysis and Hyponatremia: A Case Report

**DOI:** 10.7759/cureus.90706

**Published:** 2025-08-21

**Authors:** Anshu Solanki, Dev Mukerjee, Naveen Bhadauria, Rudra Barve

**Affiliations:** 1 Rheumatology, North Middlesex University Hospital, London, GBR; 2 Internal Medicine, St. George's University School of Medicine, St. George's, GRD

**Keywords:** creatine kinase, hyponatremia, olanzapine, rhabdomyolysis, schizophrenia

## Abstract

Olanzapine, a second-generation antipsychotic, is generally well tolerated but can rarely be associated with serious complications such as rhabdomyolysis and hyponatremia. We report the case of a woman in her early forties with a 15-year history of paranoid schizophrenia who developed bilateral foot drop and seizures in the context of severe hyponatremia and markedly elevated creatine phosphokinase (CPK) levels. Symptoms arose after olanzapine dose escalation from 10 to 20 mg daily to address difficult psychiatric symptoms. Magnetic resonance imaging (MRI) showed diffuse T2 hyperintensity in calf muscles, and nerve conduction studies revealed bilateral common peroneal neurapraxia. The presentation was attributed to olanzapine-induced syndrome of inappropriate antidiuretic hormone secretion (SIADH) and rhabdomyolysis. Both olanzapine and Lurasidone were stopped on admission; sequential re-challenge identified olanzapine as the probable causative agent. This case highlights the importance of early recognition of muscle symptoms and electrolyte disturbances in patients on antipsychotics, particularly following dose adjustments.

## Introduction

Olanzapine is a widely prescribed atypical antipsychotic for schizophrenia and bipolar disorder. While generally well tolerated, it has been linked to rare adverse events such as rhabdomyolysis, with an estimated frequency of 0.1%-0.2% among adverse drug reaction reports involving atypical antipsychotics [1,2,3]. Rhabdomyolysis involves skeletal muscle breakdown, typically identified by elevated creatine phosphokinase (CPK) levels, and can progress to acute kidney injury if untreated [4]. Hyponatremia due to SIADH is another infrequent but potentially severe complication associated with antipsychotics, capable of triggering seizures or altered mental status [1]. Reported cases suggest onset can occur after both recent initiation and long-term therapy, sometimes triggered by dose escalation [5,6]. Proposed mechanisms include direct myotoxicity, immune-mediated inflammation, and metabolic derangements [7,8].

A recent review of antipsychotic-associated rhabdomyolysis found quetiapine to be the most commonly implicated agent (*n* = 655, 27.75%), followed closely by olanzapine (*n* = 621, 26.31%) [8]. The overlap of rhabdomyolysis and SIADH is clinically important due to potential diagnostic confusion with neuroleptic malignant syndrome (NMS) and the risk of severe neurological sequelae.

We report a case of olanzapine-associated rhabdomyolysis and SIADH following therapeutic dose escalation. The case is noteworthy for delayed onset after long-term therapy, reproducibility on re‑challenge, and the development of compressive neuropathy leading to bilateral foot drop.

## Case presentation

A woman in her early forties with a 15-year history of paranoid schizophrenia was maintained on olanzapine 10 mg daily. Approximately one week after her olanzapine dose was increased from 10 to 15 mg, she began experiencing mild bilateral calf pain. The olanzapine dose was further escalated to 20 mg approximately 15 days before hospital admission. The calf pain worsened progressively over the following weeks, accompanied by increasing lower limb weakness, which ultimately led to bilateral foot drop. Her psychiatric medication history leading up to admission is summarized in Table 1. On the day of admission, she experienced a generalized tonic-clonic seizure, prompting urgent medical evaluation. 

**Table 1:** Psychiatric medication history.

Medication	Dose and frequency	Route	Duration before presentation
Olanzapine	5 mg twice daily	Oral	Since 2018
Olanzapine	5 mg twice daily → 15 mg once daily	Oral	Dose increased ~2 months prior
Lurasidone	74 mg nightly	Oral	Initiated 1 month prior
Olanzapine	15 mg once daily → 10 mg twice daily	Oral	15 days prior

On neurological examination, she had bilateral foot drop (0/5 ankle dorsiflexion, 0/5 hallux extension), high-stepping gait, and sensory deficits in the left leg, with preserved proximal strength. The patient was euvolemic, with no history of strenuous exercise, trauma, or surgery. No signs of infection were identified. Laboratory findings showed markedly elevated CPK (85,628 IU/L), eosinophilia, transaminitis, and low calcium/magnesium (Table 2). Serum and urine osmolality, along with urine sodium, were consistent with SIADH. Renal function and potassium were normal; urinalysis showed no myoglobinuria. Autoimmune workup, including myositis panel, was unremarkable. A comprehensive review of the patient’s medication history did not identify any other drugs that could have contributed to this presentation.

**Table 2:** Patient laboratory values with reference ranges. IU/L, international units per liter

Measurement	Patient values	Reference range values
Creatine phosphokinase, IU/L	85,628	26-192
Sodium, mmol/L	115	133-146
Eosinophils, x10^9^/L	0.85	0.0-0.4
Aspartate transaminase, IU/L	1,027	10-35
Alanine transaminase, IU/L	205	10-35
Albumin-adjusted calcium, mmol/L	2.10	2.20-2.60
Magnesium, mmol/L	1.07	0.70-1.00

**Figure 1:**
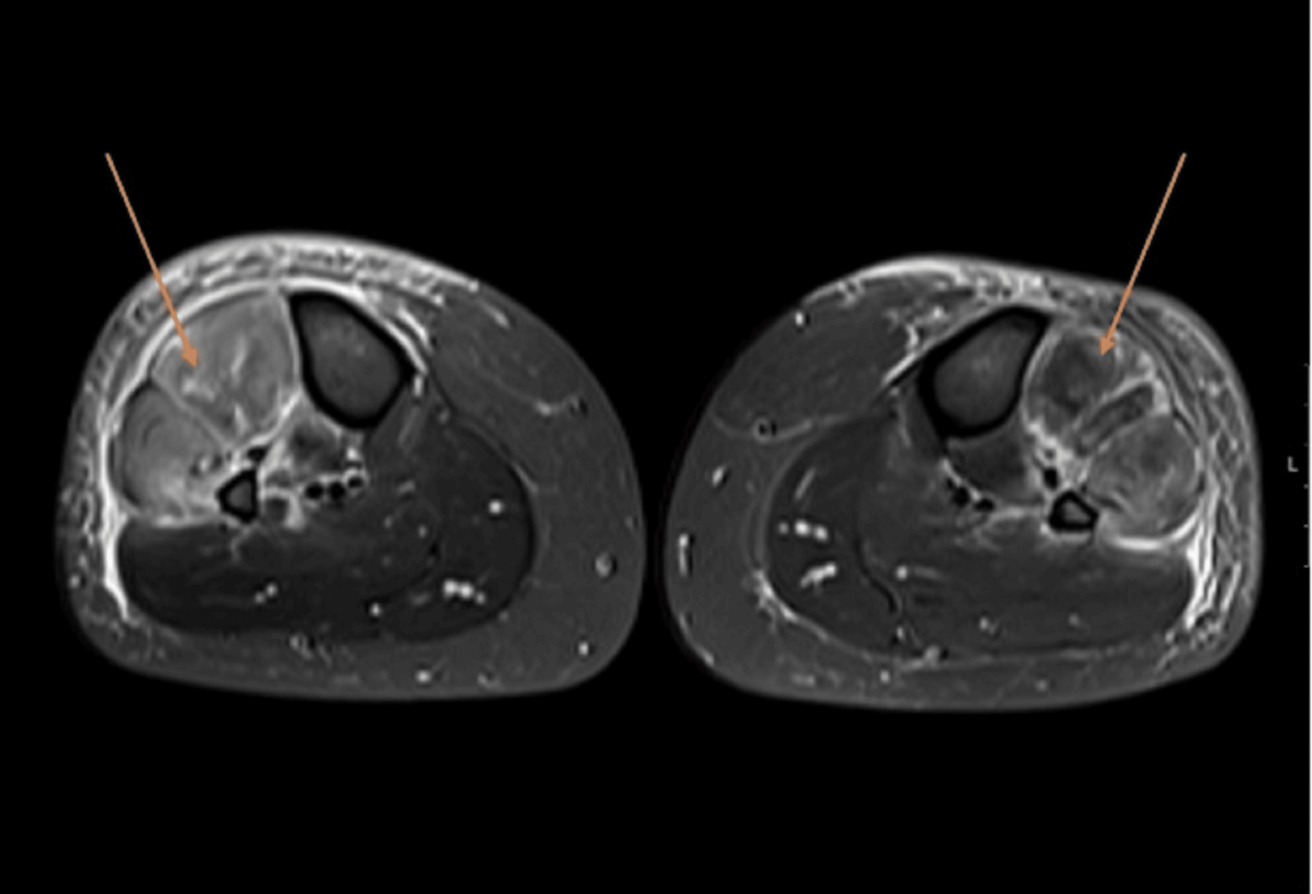
MRI calves axial view. T2 hyperintensity consistent with muscle edema (arrows). MRI, magnetic resonance imaging

**Figure 2:**
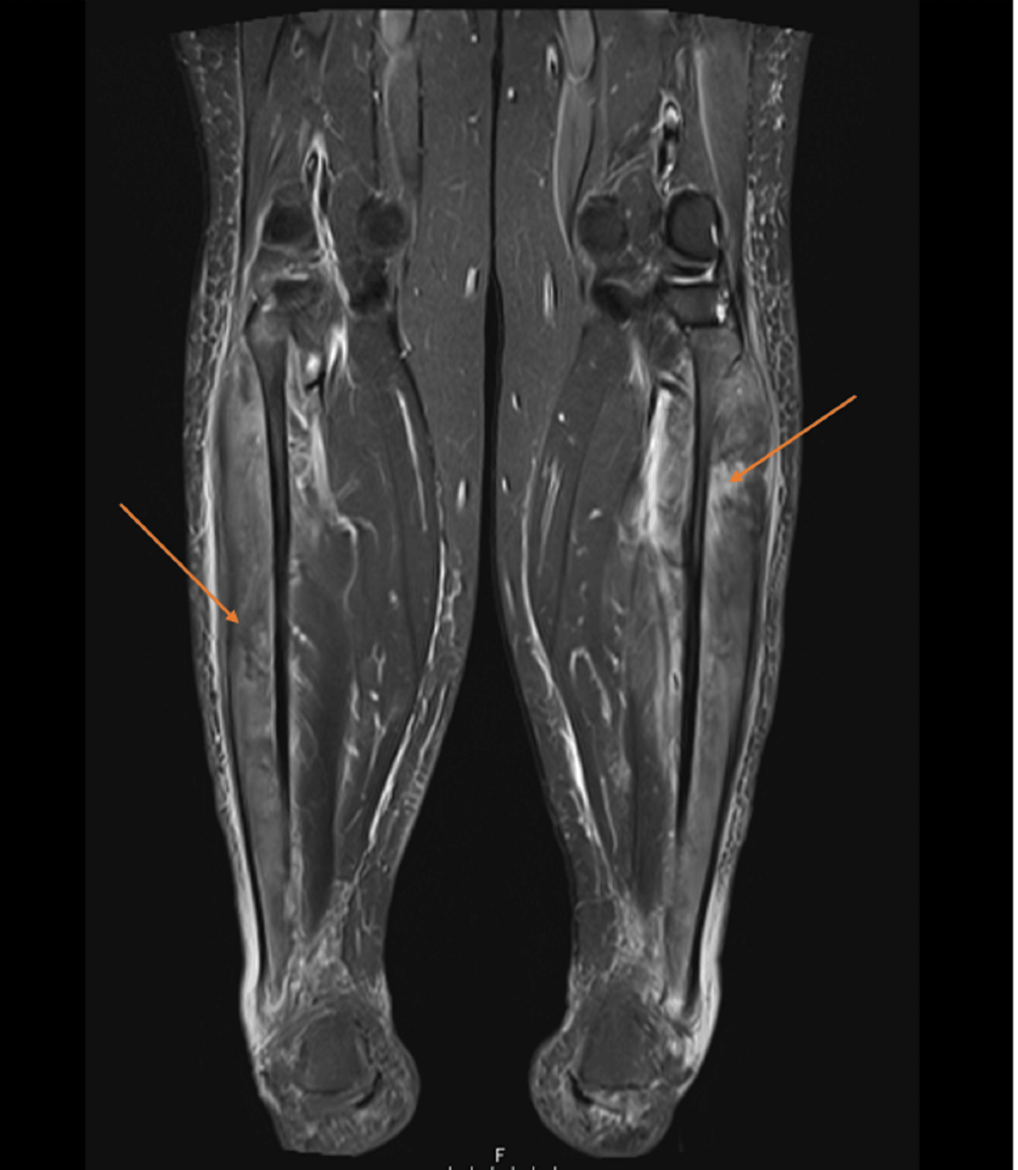
MRI calves coronal view. T2 hyperintensity consistent with muscle edema (arrows). MRI, magnetic resonance imaging

Magnetic resonance imaging (MRI) of the calves (axial view, Figure 1; coronal view, Figure 2), shown with arrows, demonstrated diffuse T2 hyperintensity with associated muscle swelling, consistent with muscle edema. Nerve conduction studies revealed bilateral common peroneal neurapraxia.

Olanzapine and lurasidone were both discontinued on day 1 of admission. The patient received intravenous saline and electrolyte correction. Once CPK had fallen to 700 IU/L, the patient was commenced on prednisolone 20 mg once daily, tapered by 5 mg every seven days until discontinuation, for possible inflammatory contribution. Improvement in symptoms and laboratory values had already begun before steroid initiation, implicating olanzapine withdrawal as the key determinant of recovery. After initial stabilization, a cautious re‑challenge with olanzapine 5 mg nightly resulted in renewed CPK elevation. In contrast, lurasidone re‑challenge produced no abnormality. Olanzapine was, therefore, identified as the probable causative drug and was permanently discontinued.

At three‑month follow-up, creatine kinase had normalized (73 IU/L), and sodium was 139 mmol/L. The patient achieved partial recovery of bilateral foot drop, with gait improvement. There was no recurrence of seizures. She was maintained on zuclopenthixol 40 mg twice daily and aripiprazole 5 mg once daily, which were well tolerated.

## Discussion

The clinical course suggests a probable adverse drug reaction: dose escalation of olanzapine led to rhabdomyolysis and SIADH; hyponatremia from SIADH precipitated seizures, while muscle swelling from rhabdomyolysis likely caused compressive neuropathy, resulting in bilateral foot drop. Although seizures can independently cause rhabdomyolysis [4], the reproducible CPK elevation on olanzapine re-challenge and the absence of such a rise with lurasidone strongly implicate olanzapine as the causative agent.

MRI findings demonstrated diffuse T2 hyperintensity and swelling, which are consistent with muscle edema, a finding seen in both inflammatory and toxic myopathies. The bilateral neurapraxia likely reflects compressive or ischemic injury related to muscle swelling, though direct neurotoxic effects cannot be excluded.

The differential diagnosis in such presentations includes neuroleptic malignant syndrome (NMS) and drug reaction with eosinophilia and systemic symptoms (DRESS). In our patient, NMS was unlikely in the absence of hyperthermia, rigidity, or autonomic instability. DRESS was improbable given the lack of systemic rash, lymphadenopathy, or multi-organ involvement. Laboratory osmolality and urinary sodium findings supported SIADH as the etiology of hyponatremia [1,4,9,10].

To strengthen objectivity, we applied the Naranjo Adverse Drug Reaction Probability Scale (Table 3), which yielded a score of 9 - consistent with a probable adverse drug reaction [11]. A re-challenge with a low dose of olanzapine (5 mg nightly) was undertaken after multidisciplinary discussion and the need to confirm causality, with close monitoring and informed consent.

**Table 3:** Naranjo score. CPK, creatine phosphokinase; MRI, magnetic resonance imaging; ADR, adverse drug reaction

Question	Response	Score
1. Prior conclusive reports?	Yes	+1
2. Event after drug given?	Yes	+2
3. Event improved on withdrawal?	Yes	+1
4. Event recurred on re‑challenge?	Yes	+2
5. Alternative causes present?	Possible (seizure)	0
6. Response to placebo?	Not applicable	0
7. Drug detected in toxic concentrations?	Not done	0
8. Event dose‑dependent?	Yes (rise after escalation/re‑challenge)	+1
9. Similar reaction in the past?	No	0
10. Event confirmed by objective tests?	Yes (CPK, MRI, electrolytes)	+2
Total = 9 (Probable ADR)		

## Conclusions

Olanzapine, even at therapeutic doses, may be associated with delayed-onset rhabdomyolysis and SIADH following dose changes. While seizure activity or other overlapping drugs may contribute, the reproducibility of CPK elevation upon olanzapine re‑challenge makes it the probable causative agent. Clinicians should remain vigilant for unexplained muscle pain, weakness, or altered mental status in patients on antipsychotics, especially soon after dose escalation. While routine CPK monitoring is not warranted, targeted testing should be considered after dose escalation or when symptoms arise. Prompt recognition, drug withdrawal, and multidisciplinary management are essential to prevent severe complications.
